# Monitoring ground water storage at mesoscale using seismic noise: 30 years of continuous observation and thermo-elastic and hydrological modeling

**DOI:** 10.1038/s41598-017-14468-9

**Published:** 2017-10-27

**Authors:** Thomas Lecocq, Laurent Longuevergne, Helle Anette Pedersen, Florent Brenguier, Klaus Stammler

**Affiliations:** 1 Royal Observatory of Belgium, Seismology-Gravimetry, Avenue circulaire 3, B1180 Brussels, Belgium; 2 Géosciences Rennes, UMR 6118, Université Rennes 1, Campus Beaulieu, 35042 Rennes Cedex, France; 3 Univ. Grenoble Alpes, Univ. Savoie Mont Blanc, CNRS, IRD, IFSTTAR, ISTerre, 1381 Rue de la Piscine, 38000 Grenoble, France; 4 BGR Federal Institute for Geosciences and Natural Resources, Geozentrum Hannover, Stilleweg 2, D-30655 Hannover, Germany

## Abstract

Groundwater is a vital freshwater resource for both humans and ecosystems. Achieving sustainable management requires a detailed knowledge of the aquifer structure and of its behavior in response to climatic and anthropogenic forcing. Traditional monitoring is carried out using piezometer networks, and recently complemented with new geophysical or satellite-based observations. These techniques survey either local (small-scale) water systems or regional areas (large scale) but, to date, adequate observation tools are lacking at the water management scale (i.e. several tens of kms), which is generally explored by modeling. Using 30 years of continuous recording by four seismic stations of the Gräfenberg Array (Germany), we demonstrate that long-term observations of velocity variations (approximately 0.01%) of surface waves can be extracted from such recordings of ocean-generated seismic noise. These small variations can be explained by changes to mechanical properties of the complex aquifer system in the top few hundred meters of the crust. The velocity changes can be interpreted as effects of temperature diffusion and water storage changes. Seismic noise recordings may become a new and valuable tool to monitor heterogeneous groundwater systems at mesoscale, in addition to existing observation methods.

## Introduction

While surface freshwater storage components (streams, lakes, snow or glaciers) are easily observable components of our landscapes, a major part of continental water resources resides below ground in groundwater systems (GW) and is broadly inaccessible to direct observations. Water flows slowly in geological layers over depths up to kilometers, locally intercepting the surface where it interacts with rivers or wetlands^1^. GW have a considerable importance for ecosystems and humanity, supporting food security and enabling adaptation to climate variability^2^. Assessing the availability of water resources requires observation techniques that can provide water storage state at different scales. The reference monitoring tool is likely to remain direct observations from water well networks, where the main challenge is data interpretation considering the relatively sparse data points with respect to the multiscale heterogeneity of aquifer systems. Complementary indirect methods are evolving to capture scale-dependent processes. Recent developments of hydrogeophysical methods^3^ or active seismic methods^4^ offer time lapse monitoring at the 100 m scale with limited vertical integration. Ground gravimetric methods are developing^5^, with potential integration at the multi-hectare scale, but are constrained by their limited sensitivity, and the difficulty and cost to carry out repeated field surveys^6^. Recent advances in space technology can provide input on subsurface water storage variations: the GRACE gravimetric mission^7^ and InSAR methods^8^ have shown their potential, but are currently limited to large (>200 000 km²) or subsiding areas. While all these methods offer potential, there is a lack of adequate tool available for monitoring ground water at intermediate scales.

In this study, we demonstrate how continuous seismic monitoring based on the analysis of ambient seismic noise can potentially fill this gap by continuously monitoring natural systems at kilometric to decakilometric scale and over time scales of tens of years. The method additionally has the potential of bringing new insights into the impact of long-term variations of precipitation and temperature on groundwater storage. Seismic noise imaging methods are based on a hypothesis of a diffuse wave field and/or wide distribution of seismic noise sources. In this case, the correlation of ground motion as recorded by two sensors will yield the “Green’s function”, or the seismic waves recorded by one of the sensors as if the seismic waves were created by a point source at the location of the other sensor. Information about the medium can be obtained through the analysis of the Green’s function even if, practically, none of these hypotheses is completely fulfilled. In particular, the combination of seismic source generation in many locations in the oceans through wave interaction and the scattering of seismic surface waves due to the heterogeneity of the Earth’s crust create a favorable situation for extracting at least parts of the Green’s function. Past research, since the ground breaking results in seismology in 2004–2005^9^ has demonstrated that fundamental mode Rayleigh waves, a specific kind of seismic surface waves, are particularly well retrieved by this method^9^, which has become standard for imaging the Earth’s crust at various scales. By analyzing the late part of the Rayleigh waves (‘coda’), i.e. the Rayleigh waves that have not followed the direct path between the sensors but rather those that have been scattered locally in the area of the sensors, the technique has been successfully extended to measure the changes of the propagation velocities over time, with applications for example to volcano and fault-zone monitoring^10,11^. In those cases, the causes of the seismic velocity changes are expected to be linked to changes in pore pressure, fluid content and the opening/closing of fractures, as these small-scale changes affect the effective mechanical properties of the medium and therefore the velocity of the seismic waves. The scope of this work is to validate seismic noise recordings as a new surface tool to capture long-term changes in subsurface water storage changes at intermediate scales. Such changes are indeed expected, as aquifers are subject to changes in saturation at shallow depth and pore pressure changes at deeper levels^12^. Additionally, aquifers are sufficiently shallow to be influenced by thermo-elastic effects^13,14^, that need to be accounted for in the hydrological interpretation. The link between small-scale changes and the effective properties of an elastic medium is known through so called homogenization techniques (e.g. Backus 1962^15^).

To demonstrate that the observed velocity changes are related to changes in the subsurface characteristics, we model the impact of surface processes (temperature diffusion, water content/pressure changes) which influence the subsurface state over a large range of time scales^13,14,16^. The modeling largely explains the observed velocity changes, and therefore strongly supports the proof of concept to use seismic noise recordings to monitor subsurface aquifers. Such modeling, when calibrated on local observations, can additionally be used to disentangle respective contributions to the observed velocity changes.

### Geological and hydrogeological setting

**Figure 1:**
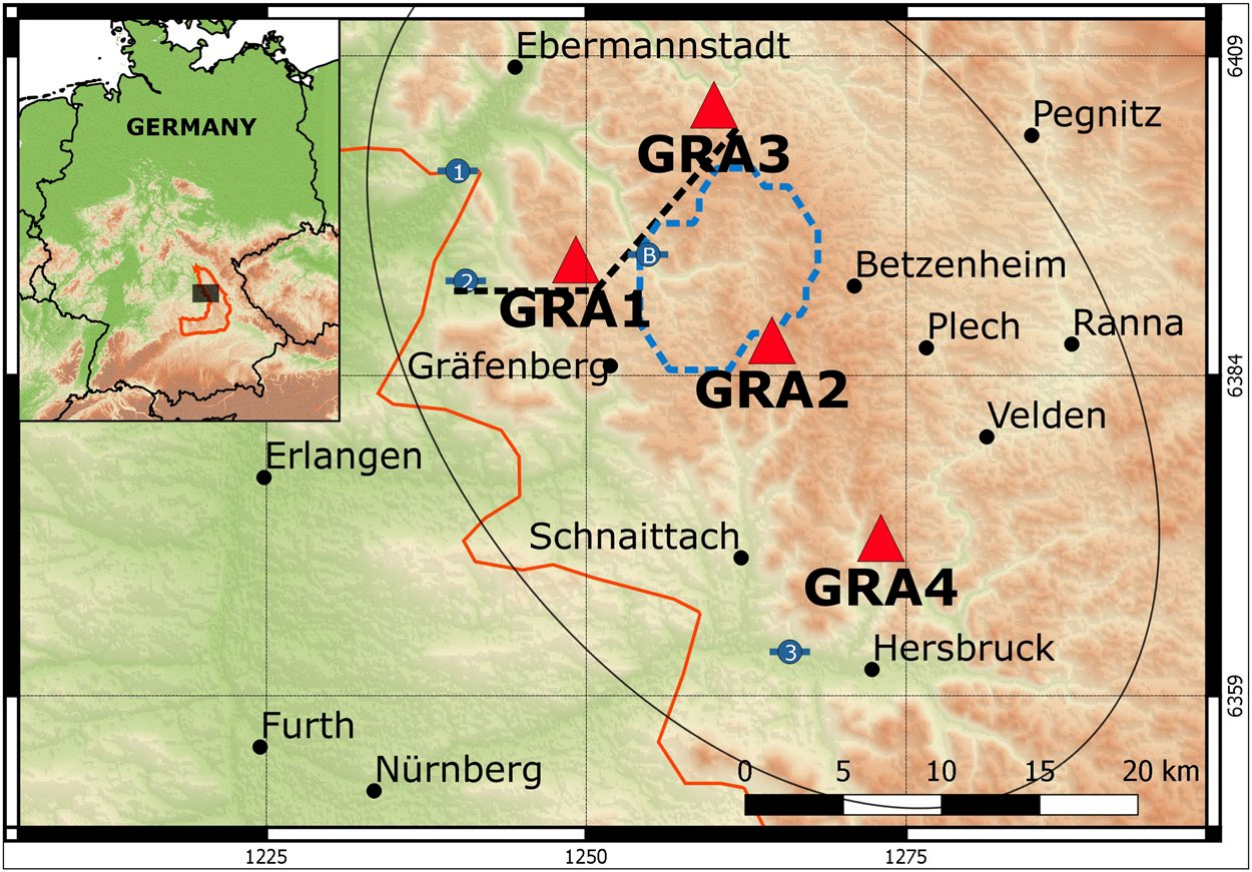
Geographical location of the study area. The GRA stations used in this study are shown as red triangles. The background topography, based on SRTM (Shuttle Radar Topography Mission^63^), is colored and shaded. The altitude ranges from 200 m to 800 m (green to brown). The piezo metric stations (1 to 3) and the Bärenthal discharge station (B) are shown as blue symbols, while the dashed blue line outlines the modeled watershed. The contour of the Franconian Jura^32^ is outlined in orange. The black dashed line shows the location of the cross-section of Fig. 2. The thin black contour shows the approximate area of maximum sensitivity of the *dv/v* measurements. The cities, coastlines and country borders are obtained from “Natural Earth data”. This map has been plotted in QGIS^61^.

The data used in this study are continuous seismic broadband records from the Gräfenberg Array in south-east Germany (Fig. 1), which is located on karstified Jurassic Limestones (Malm)^17^. This area is located north of the Alpine foreland, where the seismic hazard is considered low to moderate^18^, with no major earthquakes during the study period, and no volcanic or geothermal activity. Therefore, on the time scales considered, only processes related to surface effects (temperature, hydrological) can potentially cause changes in seismic surface waves in the area.

**Figure 2:**
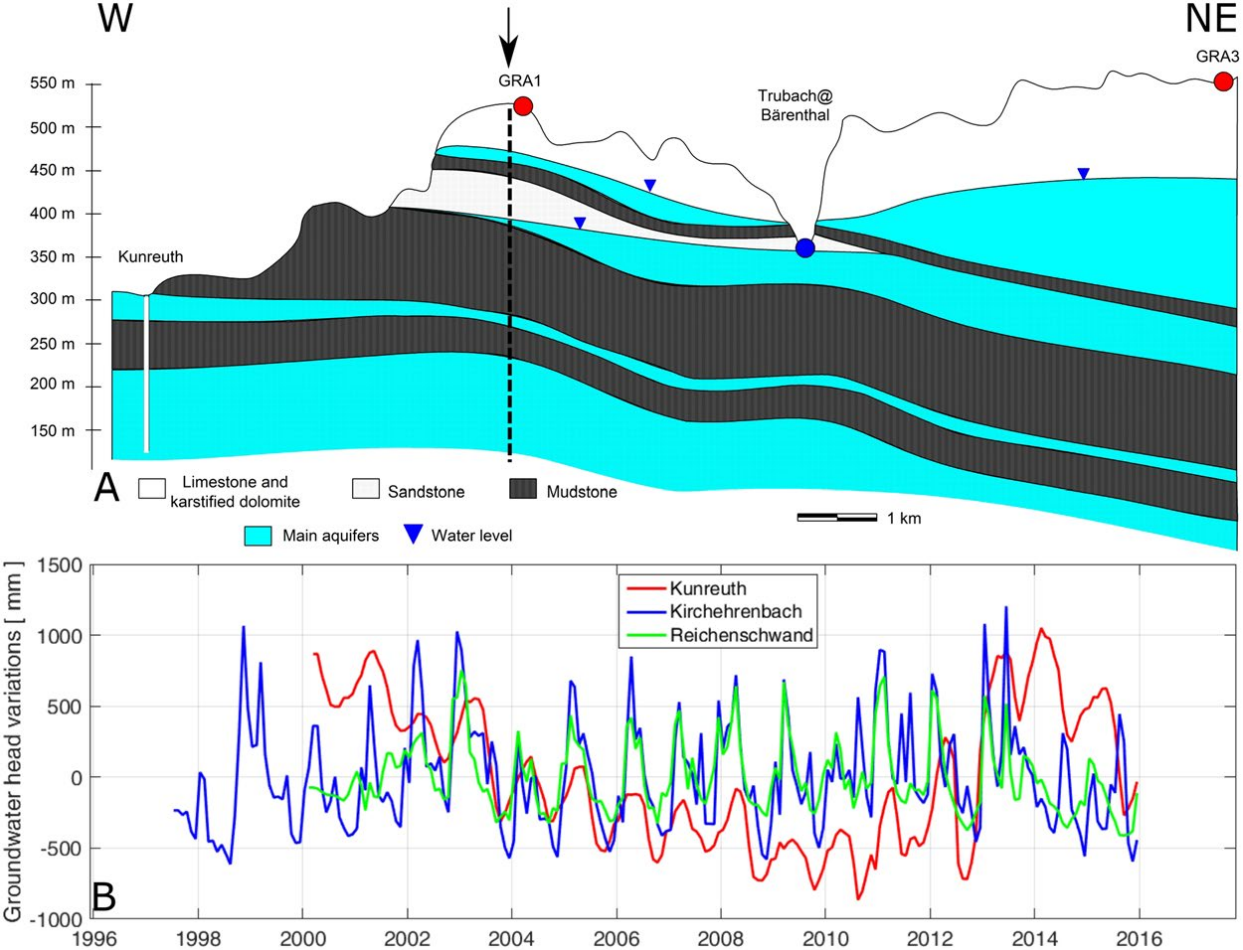
(**A**) Hydrogeological W-E cross-section (adapted from Krüger 1994^32^) illustrating the complexity of the aquifers under the Gräfenberg array (stations GRA1 and GRA3 are shown), locations of example piezometric levels (triangles) and the Bärenthal discharge station (circle) in the area. The arrow indicates where the profile changes orientation (see Fig. 1). (**B**) Groundwater head variations for the three piezometers in the area (http://www.gkd.bayern.de): Kunreuth (1 on the map in Fig. 1), Kirchehrenbach (2) and Reichenschwand (3).

The limestone layer is overlaying alternating layers of low-permeability mudstone and high permeability sandstone (Fig. 2). Both limestone and sandstone layers host significant groundwater resources, which are of prior importance in Bayern as they provide high quality water resources for a large part of the state. Both aquifers have different behavior: the shallow limestone aquifer is unconfined (i.e. water storage change translates into water level changes), while the sandstone aquifer has a confined behavior, as it is overlaid by impermeable layers (i.e. storage changes results into pressure changes, water is stored in the elasticity of the hosting rock). The Trubach river is draining both the limestone and the upper sandstone layers, the latter aquifer has therefore a mixed confined/unconfined behavior (see a simplified cross section in Fig. 2A). Groundwater fluxes are strongly influenced by topography, creating shallow (local) to deep (regional) circulation loops towards minimum topography^19^, which are modulated by the significant heterogeneities (Fig. 2) in the porous and fractured layers.

The Bavarian State office for Environment monitors three wells in the Gräfenberg region (see locations on Fig. 1, and time series on Fig. 2B): the northern and southern wells have a maximum penetration depth of 20 m, while the central one (Kunreuth) is ~180 m deep and intersects mainly the deep sandstone layers. It is therefore expected that water pressure variations in the different wells have different behavior, surface wells being more dynamic and sensitive to rainfall variations, while Kunreuth well is mainly recording slow variations associated with seasonal to interannual variability.

## Results

The Gräfenberg array was installed between 1976 and 1980, and continuous records are available for a period of forty years. We analyzed vertical component records of the first four stations to be installed for which the instrumentation was identical over 30 years, i.e. with the longest time series (GRA1, GRA2, GRA3 and GRA4; see Fig. 1). The data recorded after instrument replacement (end 2006) was not included, to exclude instrument related bias. The interstation distance is either around 10 km or 20 km (Fig. 1).

The scope of the data processing was to obtain the average long-term evolution in the study area of the relative changes *dv/v* of the coda of fundamental mode Rayleigh waves using all 6 station pairs, based on correlation of seismic noise. As the target of this study are groundwater systems, we focus the analysis on seismic noise in the second microseismic peak (around 7 s period), which in northern Europe is dominated by seismic waves created by wave interaction with the sea floor in the North Atlantic and adjoining coasts^20–22^. While using higher frequencies would increase the vertical resolution, they are dominated by non-stationary and unknown anthropogenic sources. A detailed description of the data and of the seismic noise at Gräfenberg is given the Methods section.

The data processing was carried out in three steps (for additional details, see Methods: Seismic data processing):

1. Daily noise cross-correlations were calculated using the procedure by Lecocq *et al*.^23^.
2. *dv/v*_*ijk*_ were calculated between all *k* monthly stacks for each *i-j* station pair using 80 s of coda of the cross-correlation functions starting at 20 s time lag, and the moving-window cross-spectrum method^23,24^.
3. A general inversion scheme was applied to calculate average *dv/v*_*k*_ for the whole array, then smoothed to obtain the long-term variations^25^.

The measured *dv/v* characterizes the medium in an area larger than that covered by the GRA1-GRA4 network, as the analyzed coda waves are formed by locally scattered waves. Even though the sensitivity kernel fits inside a large circular shape of 135 km radius, the use of several station pairs means that the overlapping part of the sensitivity kernels will reinforce the weight of the local structure. Note that the observed *dv/v* is bound within ± 0.01%, i.e. an order of magnitude smaller than velocity variations associated with volcanic eruptions and earthquakes^10,25,26^.

The stability of the results has been tested using two tests (see Methods: Stability testing): by removing one station (and therefore half of the station pairs) from the analysis, and by randomly selecting data points (bootstrap) for the analysis. While the details may differ between tests, all main conclusions hold.

**Figure 3:**
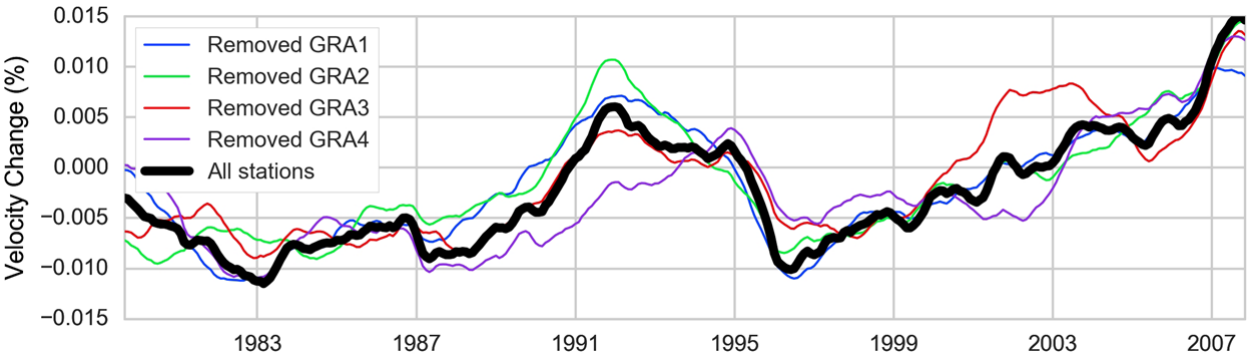
Observed relative velocity *dv/v* using all six station pairs (black) and *dv/v* calculated if data from one station is removed from the calculation (i.e. three station pairs used).

Figure 3 shows the velocity variations using the whole dataset as well as results of the test where data from individual stations are removed in turn. All the curves show consistent velocity variations with large interannual variability over the 30 years of data. The general trend is an increase of *dv/v* over the whole time period at a rate of approximately 10^−5^ per year, with small additional oscillations and with a large bulge during the 1989–1996 period. The differences between the curves of Fig. 3 indicate that it may be possible, as for volcanic eruptions and earthquakes^27–29^, to extract information about laterally varying processes within the subsurface from such noise correlations. Indeed, the lateral variability could be explained by the 3-D geometry of the aquifers, as subsurface water storage is constrained by the natural heterogeneity of geological layers.

## Discussion

On a lateral scale, the propagation path of the seismic waves is influenced by the scattering properties of the crust. To estimate an approximate area of which the observed *dv/v* are indicative, we computed and summed approximate sensitivity kernels for each the 6 station pairs^29,30^ to obtain an estimate the overall sensitivity distribution around the 6 station pairs. The ellipse shown on Fig. 1 corresponds to the 60% contour of the probability density function of the propagation paths.

On a vertical scale, the propagation velocity (phase velocity, *C*) of fundamental mode Rayleigh waves is influenced by the elastic parameters between the surface and a depth which is of the order of the wavelength, with the strongest influence located closest to the surface (down to approximately a third of the wavelength). The integration of elastic parameters over depth is not a simple average, but rather obtained through the search of null values of a relatively complex integral over depth. It is therefore not possible to finely constrain absolute velocities in the top few hundred meters with such long wavelengths. On the other hand, the velocity *changes* that we observe are likely to be dominated by changes in the uppermost layers of the Earth, as velocity changes at greater depth and over the timescales at hand are related to very active tectonic processes which are not present in the study area, such as volcanism. To illustrate how localized changes in elastic Earth parameters are related to the observed velocity changes, a forward modeling approach is required. We assume that the Rayleigh wave coda is to a first order dominated by scattered fundamental mode Rayleigh waves. Therefore, relative changes (*dC/C*) of the fundamental mode Rayleigh wave phase velocity were calculated with the CPS software package^31^ by applying localized changes in elastic parameters within a reference model for the area of GRA1^32^ (Table 1). Within this model, we applied changes in shear and compressional velocities (considering constant *Vp/Vs* = *1.73*) within a layer extending from the surface down to a given depth. The first 200 m were investigated as the lower limit for temperature diffusion at 30-year time scales, and the approximate depth of less permeable marlstone (also translated as a boundary in the reference seismic model, Table 1). Rather than using the phase velocity at a single frequency, we mimicked the observed *dv/v* over the second microseismic peak, by calculating the weighted average of the phase velocity in the period 1.2–10 s in the reference model and in the altered model. The weights were defined as the relative contribution of each frequency to the mean amplitude spectrum of all the cross correlation functions used in the study, i.e. assigning more weight to the content within the main peak between 4.5 s and 9 s period.

**Table 1:** 1D Velocity Model under GRA1, from Krüger (1994).

Layer Thickness (m)	Vp (km/s)	Vs (km/s)	Density
200	5.6 + − 0.5	3.2 + − 0.4	2.8
300	3.5 + − 1.3	0.9 + − 0.1	2.4
650	2.4 + − 0.2	1.6 + − 0.3	2.4
1000	5.9 + − 0.4	3.5 + − 0.5	2.8

**Figure 4:**
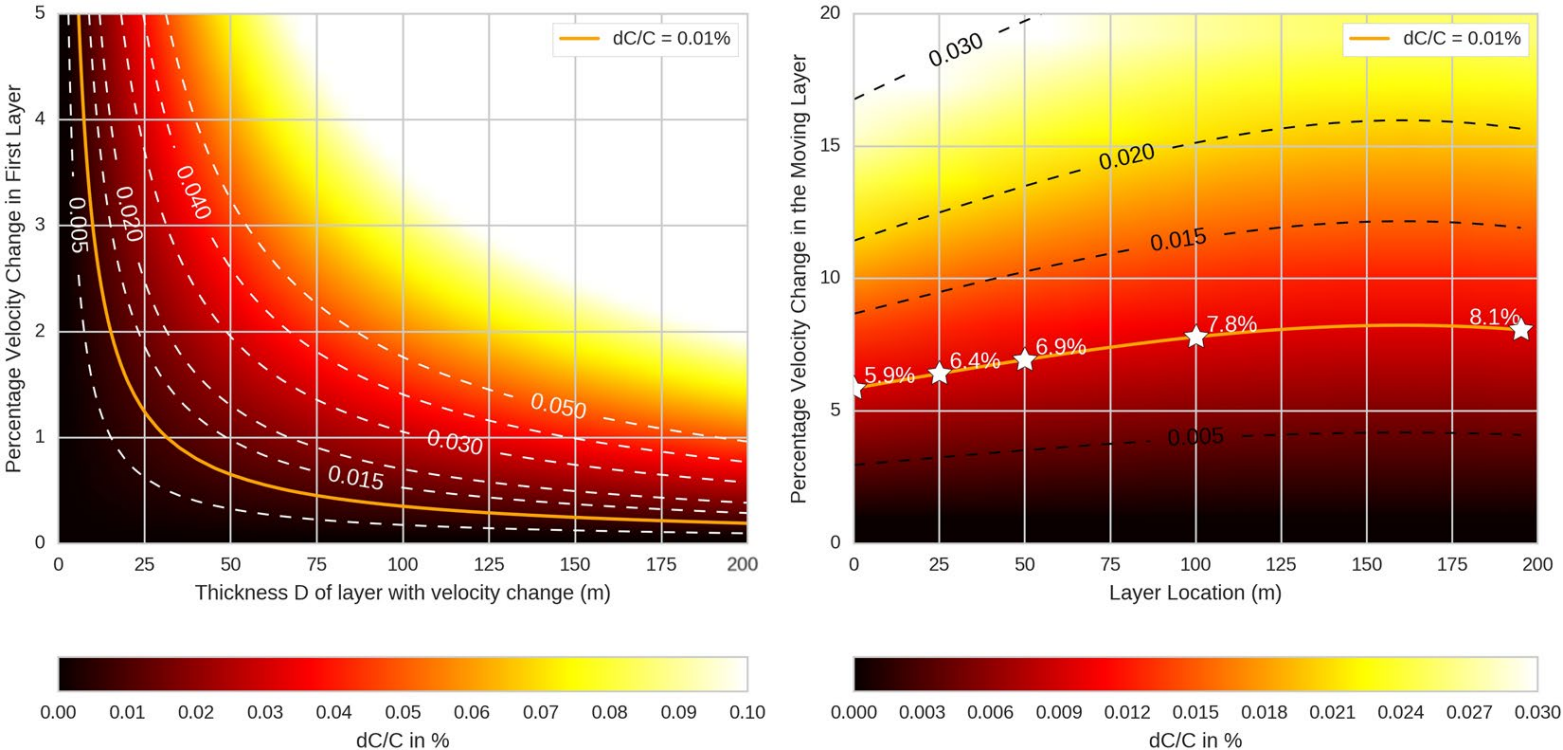
Modeled change *dC/C* (colour code) created by a change in velocity (y-axis) in: Left: a layer extending from the surface down to a depth D (x axis) or Right: a 5 meter-thick layer located at different depths. The reference model is based on Krüger^32^. The white or black dashed lines show chosen *dC/C* isolines (in %) and the orange line indicates *dC/C* at + 0.01%. The white stars on the right plot indicate example values of required P-wave velocity changes to induce an observed + 0.01% *dC/C*.

The resulting modeled *dC/C* as a function of layer thickness and velocity variation within that layer is shown on Fig. 4. The orange line shows the contour of the *dC/C* = 0.01%, which provides a target value based on the interpretation of Fig. 3. For example, a *dC/C* of + 0.01% can be produced equivalently by a + 3.0% Vp velocity change (Vs simultaneously changed to Vp/1.73) in a 10 meter thick layer or a + 0.2% Vp change in the full 200 m first layer.

With the presence of changing levels of the water table, or overpressured aquifers with varying pore pressure, the depth range of the layer with velocity change may not extend to the surface, but can be located anywhere within the depth range of the aquifer. Figure 4 therefore also shows an example of the influence of the depth of a 5 m thick layer with varying changes in elastic parameters. This calculation shows that the depth of the layer only has a minor influence on the required velocity change. For example, a 5 m thick layer located at the surface with a velocity change of +5.9% generates the same value of *dC/C* (+0.01%) as a 5 m thick layer between 195 m and 200 m depth that has a +8.1% change in velocity. Therefore, observed velocity variations can be equivalently generated by large velocity changes in localized layers (typically groundwater), or by a diffuse change throughout the subsurface layers (typically from thermo-elasticity, and for groundwater at long time scales).

The observed long-term variations in *dv/v* open exciting opportunities to monitor the evolution of subsurface systems. In this initial research, we focus the modeling on whether the observed values of *dv/v* can be related to realistic changes in elastic parameters close to the Earth’s surface, in relationship with natural processes of “external” origin. For this geodynamically stable region, the strategy adopted is to forward model the impact of both thermoelastic and hydrological contributions at interannual time scales and kilometric spatial scales. The temperature-induced changes are estimated through thermal diffusion modeling in the subsurface^13,14^, weighted by the surface wave sensitivity (see Methods).

Previous works highlighted that seismic velocities are mainly sensitive to water storage/pressure^26,33^, suggesting that the mass balance should be carefully described at the annual to decadal time scales, including the impact of climate variability. Considering the limited information on the geological layers and poroelastic parameters of the heterogeneous aquifers (Fig. 2) and the reduced number of wells to calibrate a transient flow model, we used a simple approach by setting up a water balance model at the catchment scale, which could be calibrated on river discharge and validated on available well head variations. Water storage changes are estimated based on the GR4J rainfall-runoff model^34^, using locally observed precipitation and evapotranspiration and calibrated on river runoff. Daily temperature and precipitation are obtained from E-OBS^35^, monthly potential evapotranspiration is extracted from CRU TS 3.23^36^ and river discharge and groundwater data are available on the Bavarian state office for Environment website (http://www.gkd.bayern.de). Although the model is conceptual, it has been successfully applied to represent the significant groundwater contribution to Nepal rivers^37^ (see the Methods section). Both modeling results are then translated into equivalent pressure changes. The key hypothesis behind this approach is to assume the changes at depth to be small, i.e. that a first order Taylor expansion can be used to describe the complex relationship between thermal or hydrostatic induced pressure variation and seismic velocity variation *dv/v*. Practically this means that a linear relationship (scaling) is assumed between pressure change and *dv/v*. While both effects are correlated – and difficult to separate - at annual time scales, we focus here on long-term evolution to decipher their respective contribution. We refer the reader to the Methods section for further details.

**Figure 5:**
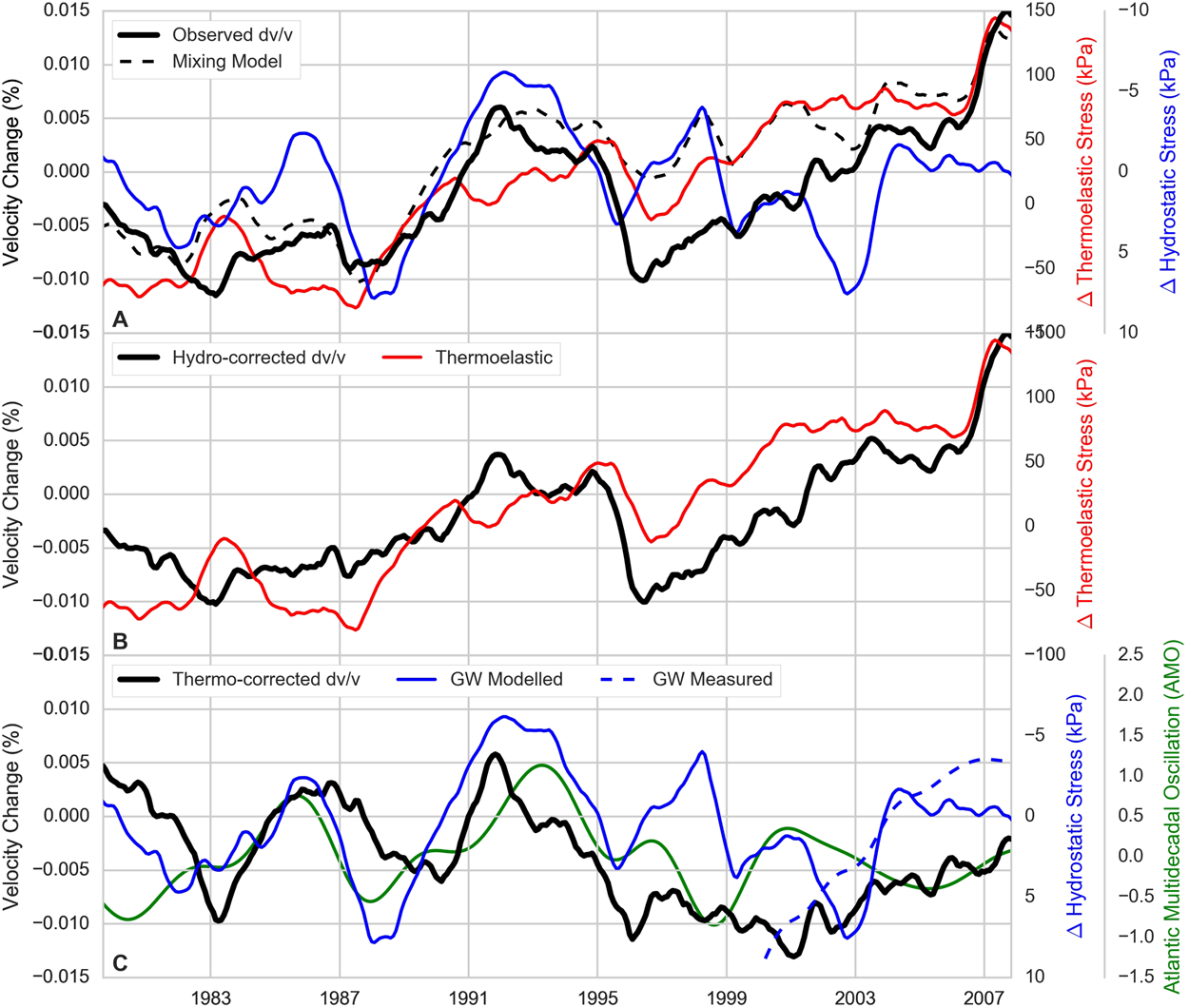
Observed *dv/v* and results of modeling. (**A**) Observed *dv/v* (black), modeled *dp*_*t*_ (red), modeled *dp*_*h*_ *(blue) and Mixing model M* = 0.5 *α.*dp*_*t*_ − 0.5 *ß.*dp*_*h*_ (dashed black); (**B**) *dv/v* + β.*dp*_*h*_ (“hydro-corrected *dv/v*”, black) and *dp*_*t*_ (red). (**C**) *dv/v* − α.*dp*_*t*_ (“thermo-corrected *dv/v*”, black), *dp*_*h*_ from model (blue) or observed (dashed blue) and Atlantic Multidecadal Oscillation index (green).

Figure 5 shows the result of the modeling, first with the observed *dv/v* and the pressure change due to thermoelastic (*dp*_*t*_) and hydrostatic (*dp*_*h*_) effects. The factor between the vertical scales (α and β) is calculated so that the standard deviation of the detrended pressure curve equals the standard deviation of detrended *dv/v* (see Methods). The velocity-pressure conversion factor α (linked to rock compliance) for thermo-elasticity is 1.76 10^−6^ Pa^−1^, which is coherent with estimates based on the analysis of tidal components^38^ or surface mass changes from ice sheet melting^33^. The fact that α is positive might be seen as counter-intuitive when considering the single impact of thermal dilatation on seismic velocity, but similar positive correlation has been found by Richter *et al*.^13^, suggesting that in terms of seismic velocities, the contribution of compressional stress changes due to the confinement of the medium dominates over the effect of thermal dilatation.

The scaling β to water pressure variations is −1.28 10^−5^ Pa^−1^, i.e. negative and ten times larger than α, which is expected in the presence of pressurized saturated rocks^39^. Figure 5C shows (*dv/v* − β.*dp*_*h*_) and (*dv/v* − α.*dp*_*t*_) in order to explore whether these two effects can explain all or part of *dv/v*, and if so, what is the relative weight of each.

Figure 5B shows that the long-term evolution of *dv/v* remarkably matches the thermoelastic effect. This is particularly true for the progressive increase since 1996 and sharp increase in 2006. Figure 5C demonstrates that the hydrological effects may well explain some of the variations of short duration that were not accounted for by the thermal effect. In particular, the 1989–1996 increase in the *dv/v* is not fully explained by thermoelastic effects but it matches the modeled decrease of pressure *dp*_*h*_ associated to a decrease in modeled groundwater storage. During this period, the European climate showed anomalous behavior with hot and dry summers in Europe. This period coincided with a substantial warming of the Atlantic Ocean^40^. There may therefore be a strong link between the Atlantic Multidecadal Oscillation, which is a major mode of climate variability^41^ and groundwater storage. Finally, the hydrological model fails to capture the 1996–2007 observed storage decrease (Fig. 5C, plain and dashed blue lines, respectively) while it is contained in the thermo-corrected *dv*/*v* obtained from the seismic records. This may be linked to many factors, including model structure deficiencies, errors in forcing data or the different spatial and vertical integration between model and observation. It is also important to note that water pumping and water use is not accounted for in this model. Water extraction data provided by the Bavarian State office for Environment is indeed limited to region-wide annual statistics over a few years, which is not sufficient to adequately represent such processes in the model. It is noticeable that the water head level observed at Kunreuth (the piezometer that samples a deep aquifer) decreases during 2001–2006, a period during which we observe a steady increase in *dv/v*. The overlap in time series is, however, too short to confirm a simple relationship between the two observations.

To decipher the respective contribution of both processes, we focus on the temporal variability as a first-order model to reconstruct observed velocity variations. It is important to note that counter to short-term and annual variations, the correlation between *dp*_*t*_ and *dp*_*h*_ is weak over 30 years (0.1), so both can add up or cancel each other depending on the succession of wet/dry and warm/cold events (Fig. 5A). Temperature and precipitation (and therefore groundwater storage) are however correlated, as can be seen during the hot and dry years of 1989–1996. Under the rather limiting approximation of taking into account only these two processes in long-term changes in *dv/v*, it is possible to estimate their relative contributions. The highest correlation coefficient (0.83) between the combined effect and observed *dv/v* occurs when thermoelastic effects count for 50% and hydrostatic effects count for 50% are considered(Fig. 5A). The 0.83 correlation coefficient drops to 0.76 (respectively 0.42) if interpretation is solely limited to the thermal effect (respectively the hydrological effect).

## Conclusions

We demonstrate the ability of long-term monitoring using ambient seismic noise to highlight modifications in mechanical properties in the near subsurface and link them to environmental and climatic external origin. The 30-year seismic observations on the Gräfenberg array are an exceptional long-term dataset to investigate long-term changes in subsurface mechanical state. Careful processing of seismic velocity variations on the 1.2 s to 10 s frequency band highlights significant changes at interannual time scales, in the order of 0.01%. The observed velocity variations can be generated equivalently by large velocity changes in localized subsurface layers (typically groundwater) or by a diffuse change throughout the subsurface layers (typically from thermo-elasticity, but also for groundwater at long time scales). These variations are attributed to an equivalent mixing of thermo-elastic (50%) and hydrological (50%) contributions, based on modeled pressure changes in the subsurface. While these results are still preliminary and three dimensional effects are not taken into account, they demonstrate that both thermo-elastic and hydrological contributions are significant and of the same order of magnitude. It is expected that velocity variations among station groups (Fig. 3) is due to spatial variability in hydrological behavior, while thermoelastic impact would be more homogenous.

Considering the availability of seismic observations, methods based on seismic noise therefore offer tremendous opportunities for the long-term monitoring of subsurface heterogeneous aquifer systems from surface observations. Future new prospects consists in analyzing 4-D images of the subsurface through the exploration of different frequencies to provide depth resolution, while lateral variations would be recovered through the use of denser seismic arrays. It is expected that such methodological improvement would lead to a finer understanding of shallow and deep groundwater flow and its relation to climate. Three-dimensional sensitivity kernels of seismic surface waves will allow, in the future, to better constrain the location of relative velocity changes and, therefore, feed new comparisons with finer hydrological models that will include strong heterogeneities like those present in the Gräfenberg area.

## Methods

### Seismic data and seismic data processing

#### Data

The data used in this study are continuous seismic broadband records from the Gräfenberg Array located in south-east Germany (11.33°E, 46.64°N), and operated by the BGR (Bundesanstalt für Geowissenschaften und Rohstoffe). The Gräfenberg array was installed between 1976 and 1980, so continuous records are available for a period a forty years and is subdivided in three sub-networks. Originally each main station (GRA1, GRB1 and GRC1) was equipped with a Streckeisen STS-1 3-component sensors^17^, while the others were equipped with STS-1 vertical component sensors. In 2006, all stations were replaced by 3-component Streckeisen STS-2 sensors. The data is sampled at 20 Hz and was originally band-passed between 0.05 Hz and 5 Hz. The Gräfenberg Array has previously been used for the array study of microseism^20^ as the anthropogenic noise is relatively low and the interstation distance of 20 km ensures that the whole array records approximately the same microseismic noise. For this study, vertical component records of four stations were analyzed: GRA1, GRA2, GRA3 and GRA4, i.e. the first four stations installed and thus the longest time series. Their location is showed on the map in the article (Fig. 1). We analyzed a 30 year long time series, as the change of equipment in 2006 might introduce instrument related bias.

#### The seismic noise at Gräfenberg

**Figure 6:**
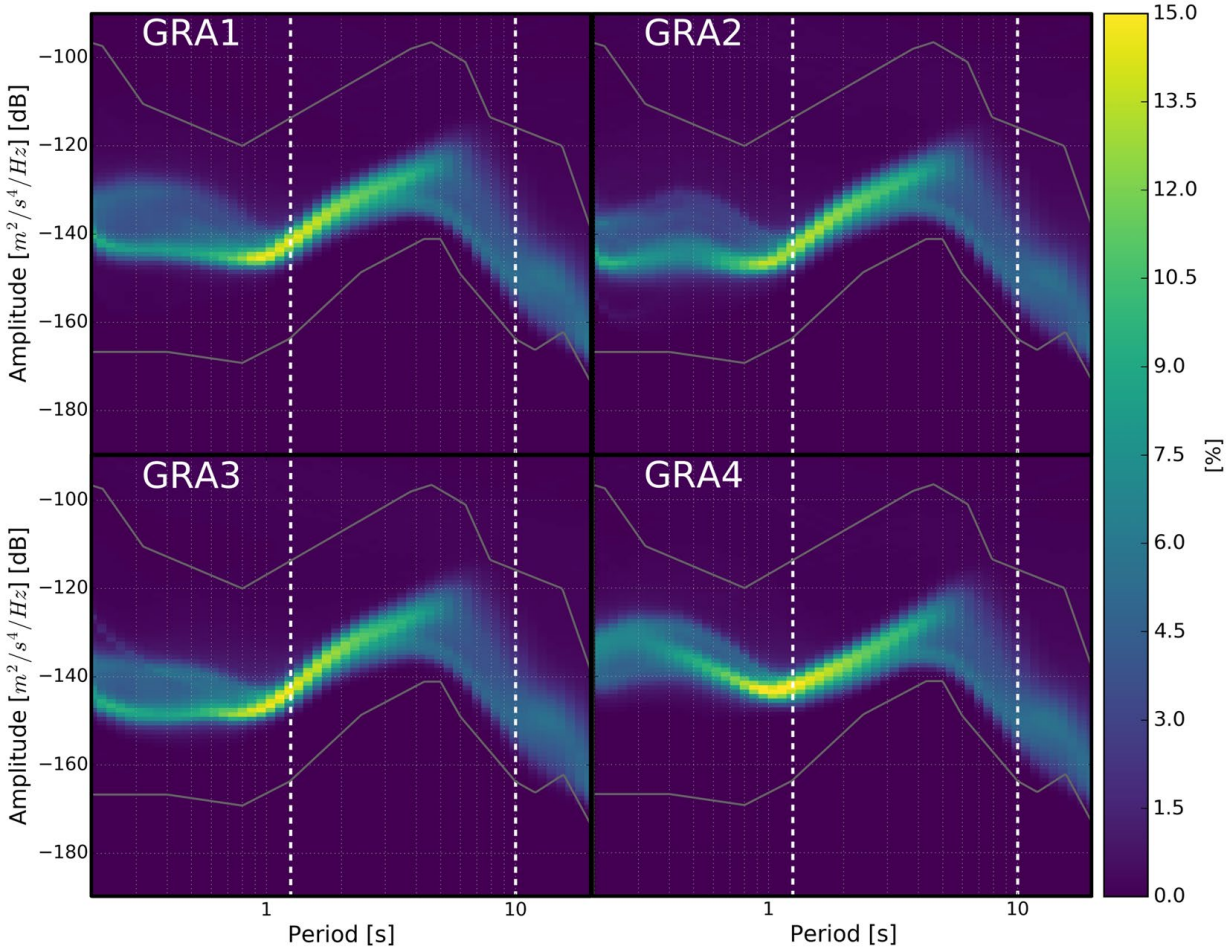
Probabilistic power density spectrum (PPSD) of the vertical component (BHZ) records from the 4 stations used in this study. PPSDs are constructed from 1 hour individual PSDs, overlapping by 50%. The time span shown here includes all data available between 1977 and 2006, i.e. more than 500.000 individual Power Spectral Density (PSD). At each pixel (i.e. given period and amplitude), the color scale shows the percentage of spectra that fall into the pixel. The data acquired by the stations has been band-passed at acquisition time between 0.05 Hz and 5.0 Hz, so only this range is presented here. The frequency band used for the *dv/v* calculation is comprised between the white dashed lines, from between 1.25 s and 10 s. The New High or Low Noise Models^43^ are shown for reference.

**Figure 7:**
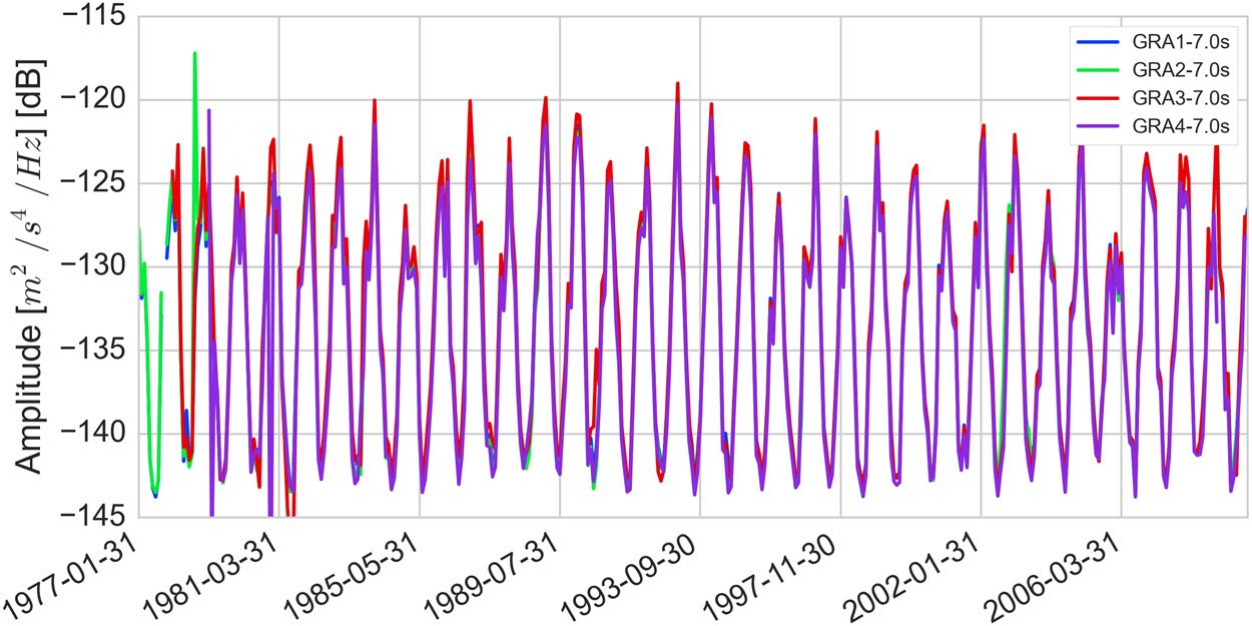
Time series of the PPSD value at 7.0 seconds extracted for the four stations (BHZ) used in the study.

The noise level at a seismic station can be evaluated using the probabilistic power spectral density (PPSD) of its records^42^. The correlation analysis targets the period band 1.25 s–10 s, in which the dominant peak is at about 7 s period. As seen in Fig. 6, the PPSD for the four stations is very similar, and locate in the medium-low level of standard noise models^43^. The noise level is very stable between periods of 0.8 s to 3.0 s. At smaller periods, the noise is dominated by anthropogenic sources. Above 1 s, natural microseismic energy dominates with a stronger seasonality seen for the longest periods: higher in the winter (see Fig. 7 for the noise level at 7 seconds), when the source of seismic noise is located in the Northern Atlantic^20–22^. Within our period band of interest, we verified that the noise level in each hour long window is approximately identical between each pair of stations (also visible from Fig. 7), confirming that the noise records are not dominated by local noise sources.

#### Reconstruction of Cross Correlation Functions from seismic noise

We computed the daily vertical-vertical noise cross-correlations functions (CCF) using a standard processing scheme^23^. As we are quantifying minute changes, any minor processing issues could influence the results; therefore we reproduce all details here. The daily records are first pre-processed:

- Check for the timing of each continuous trace and align on sampling grid if needed.

- Check gap durations, fill gaps smaller than 10 samples with interpolated values and keep the larger ones.
- Demean, taper and high-pass the traces above 0.08 Hz.
  As the instruments are identical up to 2006, it was not necessary to correct for instrument responses.
  To obtain the daily CCF, the daily records are cut in 30 minutes, 50% overlapping windows:
- Demean and taper (length of the half-width cosine taper: 36 seconds).
- Clip the amplitude of the seismograms to 3 times the RMS of the window.
- Whiten in the frequency domain between 0.1 and 1.0 Hz and add a taper on each side.
- Cross-correlate: multiply spectra, inverse FFT and normalize on the energy.
- Compute average (linear stack) CCF for one day.

**Figure 8:**
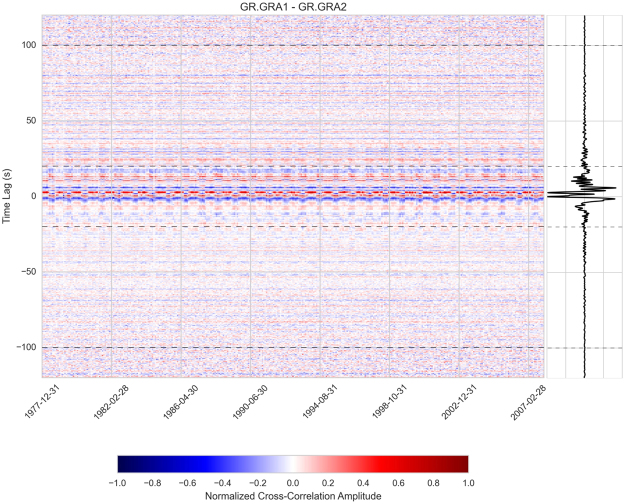
Monthly cross-correlation functions over 30 years for station pair “GRA1-GRA2”, colour coded by amplitude. The horizontal dashed lines indicate the part of the coda used in the *dv/v* calculation. The right panel shows a stack of all months.

The daily CCFs were then linearly stacked at the end of each month, including data from the previous 31 days. Monthly stacks are reliable provided further smoothing or averaging is applied. This is the case here, as we apply smoothing to extract the long-term variations. At the end of this part of the processing, we had obtained an average correlation for each month and station pair (Fig. 8).

#### Moving Window Cross Spectrum and computation of *dv/v*

To obtain a measurement of the relative velocity variation *dv/v* as a function of time (for us, 30 years), it is, as a first step, necessary to measure very small time delays in the surface wave coda. Traditionally, these time delays are obtained by comparing each “current” CCF with a “reference” CCF^10,23^, arbitrarily defined as a stack of the available CCFs, e.g. over the whole period. This comparison is carried out either in the time domain (“stretching method”) or in the frequency domain (“doublet method”). When very small changes are expected, the doublet method^44^ and its moving-window version^24^ are preferred^45^. We used the method by Brenguier *et al*.^25^ which avoids defining a reference CCF by computing all possible MWCS combinations, i.e. each monthly CCF is “compared” to all other (N) monthly CCFs, i.e. N * (N − 1)/2 calculations of *dv/v*_*ijk*_ are needed per station pair.

At short time lags, Colombi *et al*.^46^ demonstrated that *dv/v* measurements are influenced by the source. In practice, it is therefore necessary to use the coda of the CCFs to measure *dv/v*. As the interstation distance is smaller than 20 km, the ballistic surface waves arrive at approximately t = ± 3 s. To make sure that only multiply scattered waves were analyzed, we consequently used the time windows 20 s to 100 s and −20 s to −100 s. Within these limits, we used a 12 second sliding window (2 s step between each window).

In each time window, we calculated the slope of the phase difference between the CCFs (11 samples) in the 0.1 to 0.8 Hz frequency band smoothed spectrum. Prior to the Fourier Transform, the data in the window was demeaned and tapered (85%). The slope was calculated using a least squares linear regression weighted by the coherency at each point. The slope is directly related to the time shift *dt*, which was attributed to the center of the window. The linear regression additionally provided an error estimate on the slope, and therefore on *dt*.

The next step of the analysis was to calculate the average *dt/t*, based on the *dt* obtained for each window. We again used weighted least squares linear regression, the weight being the inverse of the error estimate on the individual *dt*. The line was not constrained to cross the origin, allowing it to adapt to potential clock errors as such errors result in a shift along the time axis. The outcome of this second linear regression, *dt/t* for a given month, was multiplied by −100 to obtain *dv/v* in percent^25,47^. At the end of this part of the processing, we had, for each station pair, obtained a measurement of *dv/v*_*ijk*_ for each station pair of the 360 months.

#### Inversion

Through MWCS, for each *i-j* station pair, we obtained 360*(360-1)/2 *dv/v*_*ijk*_ values. The objective of the final inversion was to calculate the average *dv/v*_*k*_ for each month across all the station pairs. The system is thus largely overdetermined. A generalized least-squares inversion^48^ is applied to solve for the model *m* (in our case, *dv/v*_*k*_ for each *k* month):

1 $$m={({G}^{t}{C}_{d}^{-1}G+\alpha {C}_{m}^{-1})}^{-1}{G}^{t}{C}_{d}^{-1}d$$

where *d* is a vector containing all values of *dv/v*_*ijk*_ and G is a sparse matrix containing the index of the days used for each *d*_*i*_ value. *C*_*d*_ is a diagonal covariance matrix of dimension $[\frac{n(n-1)}{2},\,\frac{n(n-1)}{2}]$ that describes the Gaussian uncertainties of the data vector *d*. *C*_*m*_ is an a priori covariance matrix of dimension *[n,n]* for model vector *m*. The parameter *α* is a weighting coefficient of the smoothing matrix *C*_*m*_. The values of *C*_*m*_ describe how *dv/v* for month *i* is correlated to *dv/v* for month *j*:

2 $${C}_{mij}={e}^{(-|i-j|/2\beta )}$$

where *β* is a characteristic correlation length between the model parameters fixed in our case to 36, i.e. 36 months.

**Figure 9:**
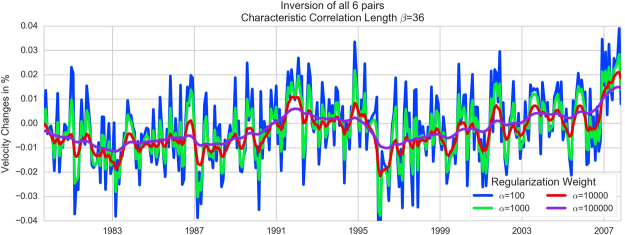
The result of the *dv/v* inversion for different regularization (smoothing) parameters α.

The smoothing parameter *α* can be adapted to highlight seasonal or more long-term trends. Values of 100, 1000, 10000 and 100000 have been tested (see Fig. 9). With *α = 100*, the yearly variations are clearly visible and are diminished to almost none with *α = 100000*, which we use to discuss the long-term trends.

#### Stability testing

In order to test the stability of the results obtained by inversion, we carried out several tests. The first one was a leave-one out or jacknife^49^, i.e. neglecting all pairs including one station and therefore reducing the amount of data information by 50% (3 stations – 3 pairs, instead of 4 stations – 6 pairs). The evolution of *dv/v* over time is stable independently of which station is excluded (Fig. 3). This is particularly true for the long-term trends. The only small discrepancy concerns the removal of the southernmost station, GRA4, specifically during the 1990–1995 period where the increase in *dv/v* occurs later when GRA4 is excluded. This effect could tentatively mean the cause of the observed *dv/v* during that period is spatially variable.

**Figure 10:**
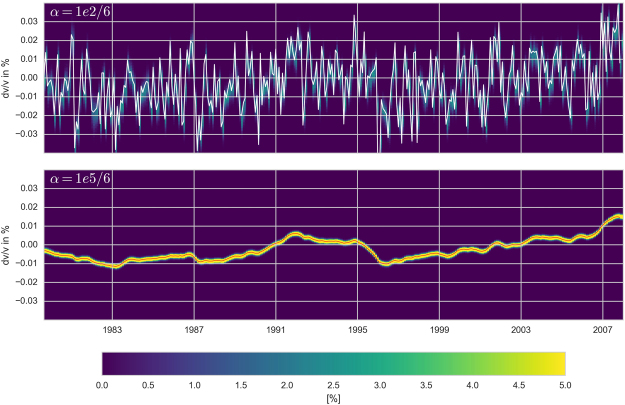
Inversion results using a bootstrap procedure. The color scale shows the percentage of spectra that fall into the *dv/v* pixel. The value computed with α = 100 (top) or 100 000 (bottom) for the complete dataset (6 pairs) are shown in white (top) or red (bottom). The use of a smaller (larger) α value induces a lighter (stronger) smoothing.

We also calculated average *dv/v*_*k*_ by a bootstrap procedure. In practice we used a single, randomly chosen, station pair for each of the 360*(360-1)/2 time windows. This procedure was repeated 5000 times, so we obtained 5000 estimates of *dv/v*_*k*_. Note that *α* was divided by 6 to take into account the reduced number of data for each inversion. We then computed, for each month, the probability density of the 5000 results. The result of this analysis is shown in Fig. 10 for two different α values. This analysis shows that, independent of “where” (which pair) the data of a single time window comes from, the final result after inversion is very stable around the mean value obtained using all pairs.

### Modeling thermoelastic pressure variations

Among the different environmental factors, thermal stress has been highlighted as a major contributor to subsurface strain variations^14,50,51^. Several strategies have been proposed to predict thermal-induced subsurface strains^52–54^, all are based on the resolution of the heat diffusion equation.

Knowledge of interannual temperature variations is key to estimate long-term seismic velocity changes, for two reasons. First, temperature evolution is known to be non-stationary, generating long-term effects on mechanical properties of the uppermost layers of the Earth. Second, the heat wave penetration depth is increasing with increasing period of this wave, leading to amplified strains in the long-term.

The modeling, which aims at providing an approximate estimate of the effect of long-term temperature variations on seismic velocities in a form comparable to the observed dv/v, has 4 steps:

### Monthly temperature variations for the Gräfenberg area

**Figure 11:**
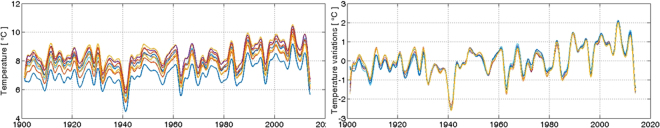
100-year temperature variations in a 100 km region surrounding Gräfenberg from CRU TS3.23^36^, after applying a 13-month Butterworth low pass filter. Left, absolute temperature variations, right, temperature variations with mean removed.

Surface temperature variations *T*_*s*_ *(t)* are extracted in a 100 km region surrounding the Gräfenberg array, from the CRU TS3.23 temperature dataset^36^, which provides ½ degree temperature analysis globally at monthly time scale for 1901–2014. While the amplitude of sub-annual and annual surface temperature variations might vary quickly at the surface, long-term variations have a quasi-homogeneous behavior over wide regions in the Gräfenberg region (Fig. 11). Furthermore, temperature changes include large-amplitude variability at 5-year to long-term time scales. As a consequence, an accurate representation of temporal variability is considered as more important than the spatial variability. We use the monthly temperature average for the region as input for the modeling.

### Temperature variations as a function of time and depth

As a 1D approximation has been shown to be suited to model vertical strains^54^, temperature variations as a function of time and depth are estimated by solving the 1D thermal diffusion equation in a homogeneous half-space $z\ge 0$,

3 $$\frac{\partial T}{\partial t}\,=\,k\frac{{\partial }^{2}T}{\partial {z}^{2}},$$

with boundary surface temperature ${T}_{s}\,(t)$ applied along the surface *z* = 0 and vanishing as $z\,\to +\infty$. *T* is temperature and *k* is subsurface thermal diffusivity. It is solved using an explicit finite difference scheme, ensuring $k\,\frac{{\rm{\Delta }}t}{{\rm{\Delta }}{x}^{2}}\le \,0.25$ for stability. We here use k = 10^−6^ m²s^−1^ ^55^.

**Figure 12:**
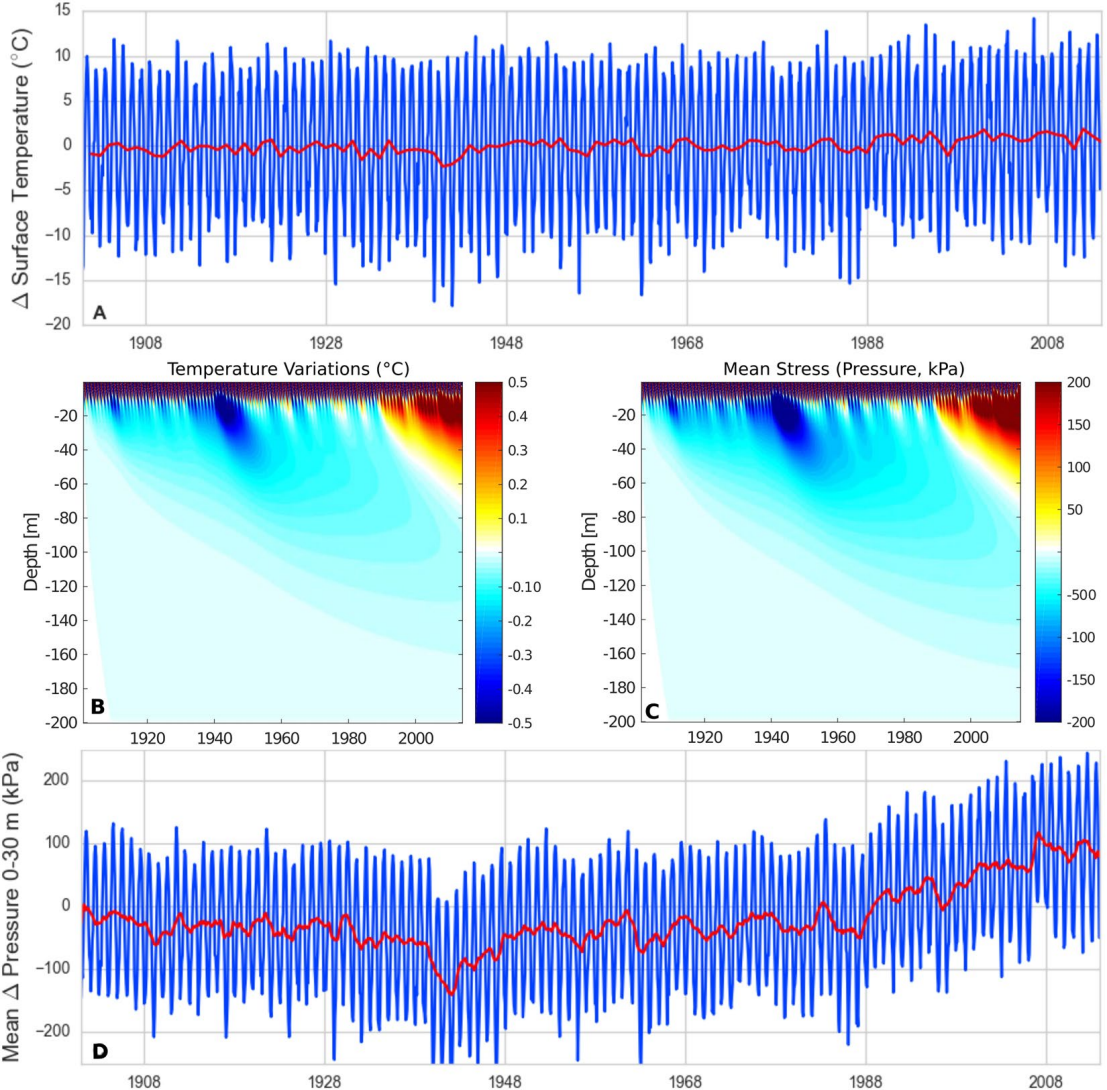
Thermoelastic stress processing scheme: (**A**) observed monthly (blue) and annual (red) mean surface temperature variations; (**B**) modeled temperature diffusion and (**C**) associated thermoelastic pressure change as a function of depth and time. The colorscale of B is saturated to highlight deep temperature diffusion. (**D**) Modeled average pressure change (blue). The long-term variation (red) is calculated as 12-month running mean and is reported as the modeled relative thermoelastic pressure variation dp_t_.

Annual temperature variations are penetrating the first 10 m of the subsurface as expected (Fig. 12). At longer time scales, though, small amplitude temperature steps (e.g. in the 1940s) are penetrating to larger depth (up to several tens of meters) and remain present in the subsurface over several tens of years. Consequently, the long-term effects have a larger influence than the annual ones on the average deformation and additionally, the impact is significant over several years, even if surface temperature decreases to its previous level. Modeled mean stresses, which are similar in magnitude to those of Ben-Zion and Allam^50^ are superimposed on observed velocity variations (Fig. 12), highlighting a positive correlation during the steep velocity increase in the 1990s and 2006.

### Pressure variation associated with the temperature variation

Considering a uniform linear isotropic elastic medium, the general stress (σ_i_)-strain (ε_i_) relationship including expansion linked to temperature change Δ*T* can be written as:

4 $$\{\begin{array}{rcl}{\varepsilon }_{1} & = & \frac{1}{E}({\sigma }_{1}\,-\upsilon \,{\sigma }_{2}\,-\upsilon \,{\sigma }_{3})\,-\alpha \,{\rm{\Delta }}T\\ {\varepsilon }_{2} & = & \frac{1}{E}(-\upsilon \,{\sigma }_{1}\,+\,\,{\sigma }_{2}\,-\upsilon \,{\sigma }_{3})\,-\alpha \,{\rm{\Delta }}T\\ {\varepsilon }_{3} & = & \frac{1}{E}(-\upsilon \,{\sigma }_{1}\,-\upsilon \,{\sigma }_{2}\,+\,\,{\sigma }_{3})\,-\alpha \,{\rm{\Delta }}T\end{array}$$

With α the linear expansion coefficient, ν the Poisson coefficient and E the Young Modulus. The half space is considered as confined in the horizontal directions, so ε_1_ = ε_2_ = 0, vertical stress σ_3_ = 0. The strategy is here to link seismic velocity change to mean stress (pressure, *p*) with

5 $$p=\frac{1}{3}({\sigma }_{1}+{\sigma }_{2}+{\sigma }_{3})=\frac{E}{3\,(1-\upsilon )}\alpha {\rm{\Delta }}T.$$

Using k = 10^−6^ m²s^−1^ and α = 10^−5^ °C^−1^ ^55^, ν = 0.26 and E = 70 GPa from reference seismic model^32^ using

6 $$\upsilon =\frac{{V}_{p}^{2}-2{V}_{s}^{2}}{2({V}_{p}^{2}-{V}_{s}^{2})}$$

and

7 $$E=\frac{\rho \,{V}_{s}^{2}\,(3\,{V}_{p}^{2}-4{V}_{s}^{2})}{{V}_{p}^{2}-{V}_{s}^{2}},$$

we obtain a conversion factor of 315 kPa/°C between p and ΔT. The scaling factor is applied to the diffused temperature field to obtain the pressure field (Fig. 12B).

### Average pressure changes as a function of time

Averaged pressure is finally estimated as the vertical integral of mean stress, weighted by the sensitivity function defined previously (Fig. 4). Considering that the pressure variations are very small, one can expect to a first approximation a linear relationship between pressure variations and seismic velocity variations, i.e. have a constant scaling factor between them.

Temperature increase leads to thermal dilation in the vertical direction. While the effect of this dilatation, expected to decrease the seismic velocities, it is counteracted by the confinement in the horizontal directions, due to which compressional stress increases. As previously observed by Richter *et al*.^13^, our modeling shows (Fig. 5) that *dv/v* is overall positively correlated with the temperature induced stress, demonstrating that the effect of confinement dominates over thermal dilatation.

### Modeling hydrostatic pressure variations

While hydrological changes has been pointed out as a potential contributor to seismic velocity variations as observed by seismic noise^56^, validation has been difficult considering the large integration (vertically and in space) of this method with respect to ground observations, and the high correlation between mixed natural processes at seasonal time scale. The strategy is here to use an independent modeling approach to constrain water storage changes at basin scale

8 $$\frac{dS}{dt}=P-E-R$$

where S, P, E and R respectively stand for water storage, precipitation, actual evapotranspiration and river runoff. On a hydrological point of view, river runoff defines the response of the catchment to boundary conditions (P, E) and represents by essence the behavior of the main storage compartments (surface, unsaturated zone, shallow and deep saturated zones), at spatial scales equivalent to the spatial integration of seismic velocity variations. Runoff is varying with high dynamics over several orders of magnitude, highlighting the response time of different storage compartments, from (fast) surface to (slow) groundwater^37,57^.

**Figure 13:**
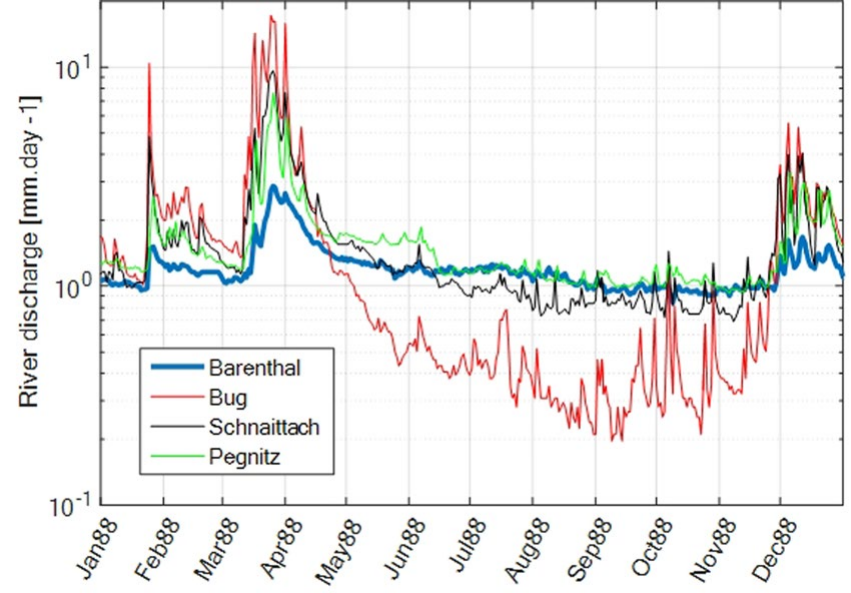
River discharge data for four discharge stations, for year 1988. Discharge can vary over several orders of magnitude, highlighting the integrated behavior of the catchment with respect to boundary conditions (P, E). Discharge variability in Bärenthal (blue line) is the smallest, suggesting a stronger control of groundwater to discharge.

We considered four discharge stations draining the Gräfenberg area: Bärenthal/Trubach river (station 24249009) located in the north, Büg/Schwabach (station 24238002) and Schnaittard (station 24228009) located in the south, and Güntersthal/Pegnitz (station 24222002) located in the east, with draining areas of respectively 61 km², 80 km², 62 km² and 318 km² (Fig. 13). Discharge data is available on the website of the Bavarian State office for Environment (http://www.gkd.bayern.de). Considering the large integration of seismic velocity variations, ground water processes are expected to dominate over the strongly time variable surface processes. Therefore, we use data from the station, Bärenthal, which best represents groundwater storage changes, i.e. with the smallest temporal variability. The model is validated using long-term groundwater data monitored in station Kunreuth (no 5179), outside the modeled catchment.

Precipitation data is provided by E-OBS^35^, which is available at a ¼ degree resolution at daily time scales from 1950 to 2015. Potential evapotranspiration is extracted from CRU TS 3.23 dataset^36^. The Atlantic Multidecadal Oscillation index is downloaded from http://www.esrl.noaa.gov/psd/data/timeseries/AMO/ ^58^.

In order to estimate groundwater storage changes, we used the GR4J rainfall-runoff model^34^, driven by observed P and E, calibrated on R. The model contains two storage components, one “production” store, which behavior is similar to shallow layers (interception of precipitation, partitioning rainfall into evapotranspiration, runoff and infiltration) and a “routing” store mimicking deep layer behavior (slow storage, contribution to river base flow). Although the model is conceptual, it has been successfully applied to represent the significant groundwater contribution to Nepal rivers^37^. The model is calibrated using a Marquard-Levenberg least-square approach on the logarithm of water discharge to limit the impact of floods and promote the description of low flows (groundwater contribution). We also add a volumetric constraint, imposing that mean modeled and observed discharge should agree within 2%. RMS error is small, only 20% of the discharge standard deviation.

**Figure 14:**
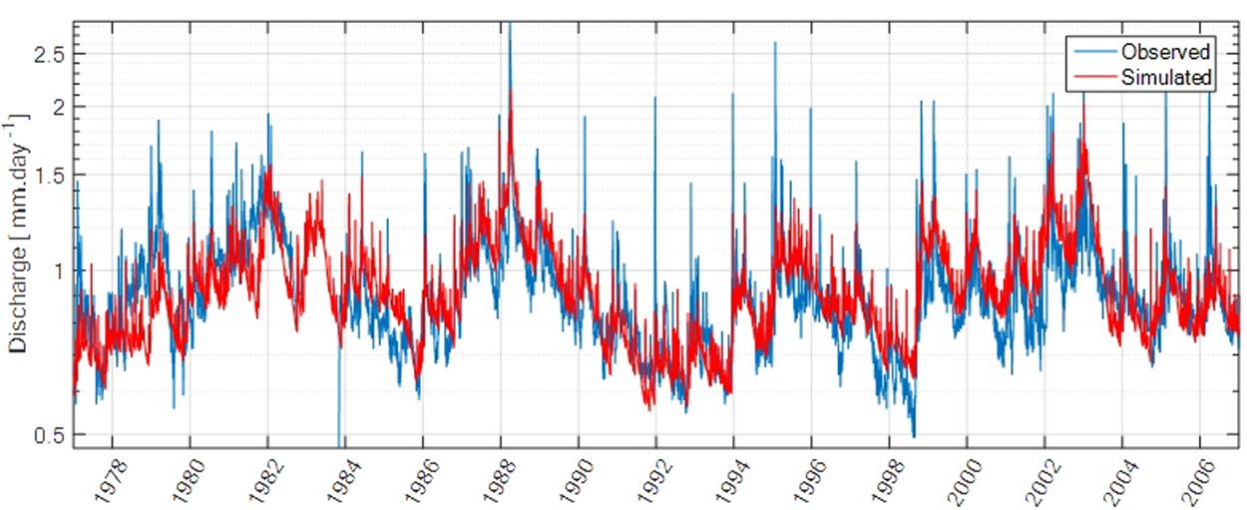
Comparison between observed and modeled discharge over the period of interest.

Modeled discharge does not represent well floods, though, the long-term evolution of base flow, including its significant interannual variations are well reproduced by this simple model (Fig. 14).

Water storage change in the “routing” store is here used to compare to seismic velocity variations and named “groundwater storage variations”. Modeled groundwater storage is transformed into pressure head to be compared to groundwater pressure observations (Fig. 5C) considering an apparent 3% porosity, which is coherent with the hosting limestone aquifer^23,58–62^.
